# Confinement-Induced Fabrication of Liquid Crystalline Polymeric Fibers

**DOI:** 10.3390/molecules27175639

**Published:** 2022-09-01

**Authors:** Jae Gwang Kim, Jae Gyeong Lee, Jeong Jae Wie

**Affiliations:** 1 Department of Polymer Science and Engineering, Inha University, Incheon 22212, Korea; 2 Program in Environmental and Polymer Engineering, Inha University, Incheon 22212, Korea; 3 Department of Organic and Nano Engineering, Hanyang University, Seoul 04763, Korea

**Keywords:** liquid crystal, liquid crystal polymers, confinement, fibers

## Abstract

In aqueous media, liquid crystalline droplets typically form spherical shapes in order to minimize surface energy. Recently, non-spherical geometry has been reported using molecular self-assembly of surfactant-stabilized liquid crystalline oligomers, resulting in branched and randomly oriented filamentous networks. In this study, we report a polymerization of liquid crystalline polymeric fibers within a micro-mold. When liquid crystal oligomers are polymerized in freely suspended aqueous media, curvilinear and randomly networked filaments are obtained. When reactive liquid crystalline monomers are oligomerized in a micro-channel, however, highly aligned linear fibers are polymerized. Within a top-down microfabricated mold, a bottom-up molecular assembly was successfully achieved in a controlled manner by micro-confinement, suggesting a unique opportunity for the programming architecture of materials via a hybrid approach.

## 1. Introduction

Liquid crystalline polymers have been a point of great interest in soft actuators and soft robotic applications [1]. In liquid crystal molecules, they are composed of mesogenic units and spacer units. In mesogenic units, anisotropic rod-like rigid benzene rings are able to induce intermolecular π-π interactions. As π-π interactions are directional attractive forces, the presence of only mesogenic units results in a crystal phase relative to the materials [2,3]. Conversely, the presence of only aliphatic spacer units tends to result in a liquid phase. Thus, it is plausible that materials could remain in a liquid crystalline phase with an appropriate balance between mesogenic and spacer units. Since liquid crystalline molecules have both segmental mobility and directional attractive forces, liquid crystalline materials would have the capacity for molecular self-assembly.

The ability of liquid crystalline materials in self-assembling provides programmability of molecular orientation. Due to this molecular programmability, liquid crystalline molecules have been utilized in various applications, one of the most successful commercialized products being liquid crystal displays (LCDs). In LCDs, twisted nematic (TN) geometry has often been employed where liquid crystal molecules have orthogonality between the top and bottom layers with a 90° twist through the thickness direction. Since liquid crystalline molecules are able to self-assemble between two programmed command surfaces, programming is only required in order for the surfaces to construct this TN molecular geometry; it is not necessary to program individual molecules. In addition, to follow the molecular direction at the interface of command surfaces, liquid crystal molecules gradually rotate 90° between the two layers. Despite the programmability of the molecular alignment, liquid crystal molecules have liquid-like mobility such that TN geometry is not permanent. When terminal groups of liquid crystalline molecules are functionalized (i.e., acrylates), however, the programmed geometry of liquid crystal molecules can be solidified via polymerization and thereby become fixed. For polymerization, photopolymerization is often adopted to maintain directional molecular geometry, as thermal energy causes random directional Brownian motions for thermal polymerization. Through self-assembly and photopolymerization of liquid crystalline monomers, liquid crystalline polymers with TN-derivatives have been utilized in various soft actuators and soft robotics [4,5,6,7,8,9,10,11,12,13,14,15,16,17,18].

When liquid crystalline molecules are freely dispersed in hydrophilic media (i.e., deionized water) without any command surfaces, their phase is separated from the hydrophilic media and forms spherical droplets. As liquid crystals mainly consist of hydrocarbons, they become hydrophobic and self-assemble with each other in order to minimize contact areas with hydrophilic media. To minimize surface tension, spherical micelles have been widely reported for the geometry of liquid crystals and liquid crystalline polymer droplets [19,20,21,22].

Recently, Shu Yang et al. reported the synthesis of fibrous liquid crystalline polymers in an aqueous solution with the presence of surfactants [23]. As is well known, surfactants have both hydrophilic head groups and hydrophobic tail groups. The addition of surfactants decreases the surface tension of water until the critical micelle concentration (CMC) is reached. Above the CMC, surface tensions no longer change by forming spherical micelles. Within the micelle, hydrophobic tail groups self-assemble at the core, surrounded by hydrophilic head groups, which are already in contact with water media. Similarly, hydrophobic liquid crystal molecules are solubilized within the various shape of micelles and self-assemble with each other, balancing between surface tension and internal elastic energy from liquid crystal molecules. At high temperatures, the micelle presented spherical shapes above the CMC because surface tension is larger than the bulk elastic energy of liquid crystal molecules. Upon cooling, however, the balance between surface tension and the elastic energy of the bulk becomes destabilized, facilitating the spontaneous formation of branched filamentous liquid crystalline monomer micelles.

Herein, we report a polymerization of highly aligned fibrous liquid crystalline polymers within a micro-channel. To prevent random directional fibril-like geometry via bottom-up self-assembly, we confined liquid crystalline monomers within a top-down micro-fabricated channel. This hybrid approach has the potential to program the architecture of liquid crystalline materials to obtain non-conventional, highly ordered fibril-like polymers without a high-shear spinning process.

## 2. Results and Discussion

**Figure 1:**
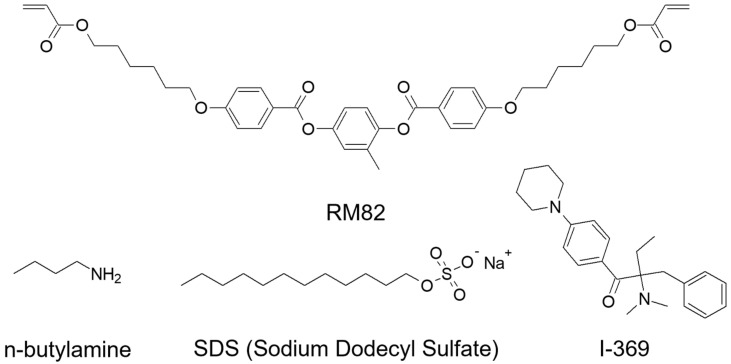
Molecular structures of chemical components were utilized in this study. 1,4-bis-[4-(6-acryloyloxyhexyloxy)benzoyloxy]-2-methylbenzene (RM82), n-butylamine, sodium dodecyl sulfate (SDS), and 2-benzyl-2-dimethylamino-1-(4-morpholinophenyl)-butanone-1 (I-369).

Liquid crystalline emulsions were prepared by mixing liquid crystalline monomers (1,4-bis-[4-(6-acryloyloxyhexyloxy)benzoyloxy]-2-methylbenzene; RM82; Synthon Chemicals), chain extenders (n-butylamine; Sigma Aldrich), and photo-initiators (2-Benzyl-2-dimethylamino-1-(4-morpholinophenyl)-butanone-1; I-369; Ciba). The chemical structures of materials used in this study are shown in Figure 1. The liquid crystalline monomer and chain extender are first dissolved in chloroform. Then, liquid crystalline emulsions were constructed by mixing the solution in aqueous media with the presence of surfactants (sodium dodecyl sulfate; SDS; Sigma Aldrich). Due to the presence of surfactants, spherical liquid crystalline micelles are formed in the emulsion, generating a gradient of elastic energy density in the radial direction of the spherical micelles. Here, the core has higher elastic energies than that of the outer part. At this spherical micelle, interfacial tension, γ, is higher than bulk director elastic energy [23].

**Figure 2:**
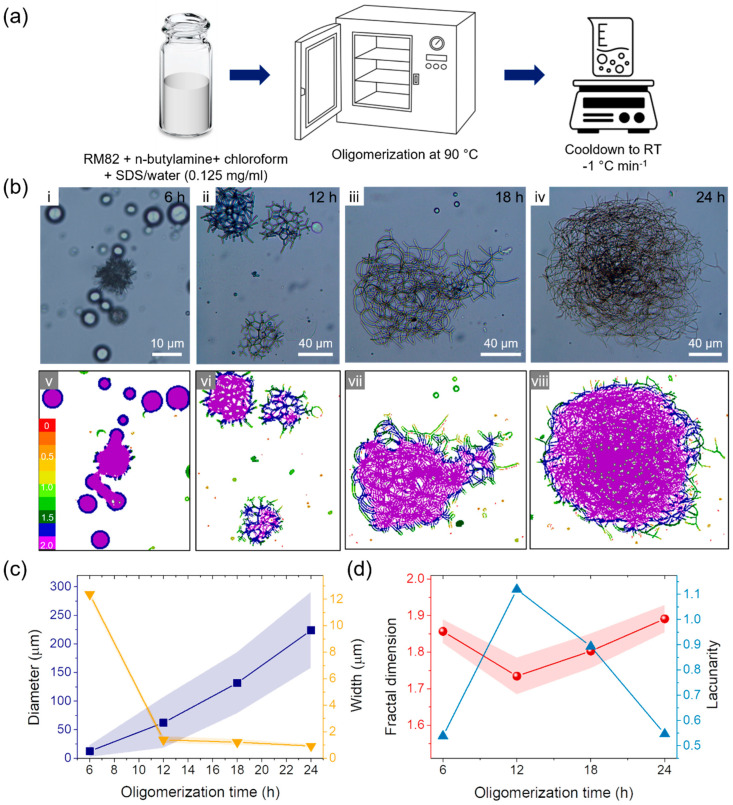
Shape transition of liquid crystalline oligomers by thermal oligomerization. (**a**) Schematic images of thermal oligomerization procedure. (**b**) Shape transition of liquid crystalline oligomers from spherical to branched and fibrous shapes visualized by optical microscopy (**i**–**iv**) and local connected fractal analysis (**v**–**viii**) against thermal oligomerization time of (**i**,**v**) 6 h, (**ii**,**vi**) 12 h, (**iii**,**vii**) 18 h, and (**iv**,**viii**) 24 h. (**c**) Diameter (■) and width (▼) of nematic liquid crystalline oligomers against thermal oligomerization time. (**d**) Fractal dimension (●) and lacunarity (▲) of nematic liquid crystalline oligomers against thermal oligomerization time.

The RM82 liquid crystalline monomer has diacrylate functional groups and is thermally oligomerized with n-butyl amine by Michael addition reaction [24] in the micelle. With a longer total oligomerization time, the mean oligomer chain length, <ℓ>, will increase (Figure 2a), and the elastic constant also increases following increases in molecular lengths [25]. The longer <ℓ> with a higher elastic constant not only increases molecular ordering but also extends the hydrophobicity of oligomer chains [26]. As molecules became thermally oligomerized, the longer oligomers migrated from the core to the outer interfaces in order to reduce the energy density gradient and grew highly ordered at the outer interfaces. Upon slow cooling, the order parameter of liquid crystal molecules increases, which can increase the number of defects at the boundary between nematic phases. This, in turn, reduces the γ [27,28]. This reduction in interfacial tension generates new interfaces, which initiates the geometrical transition from an isotropic sphere to fiber for micelles. 

Until 6 h of thermal oligomerization, liquid crystalline micelles maintain a spherical shape due to the short <ℓ> (Figure S1a,b and Figure 2b–i). Spherical micelles are known to have 2–50 nm in diameter when the thermodynamic mechanism is dominant. Spherical micelles can further grow into larger lamellar structures. When the curvature is generated in a high aspect-ratio lamellar by kinetical fluctuation, various-sized micron-scale vesicles can be formed [29,30]. In our approach, liquid crystalline micelles are kinetic-driven vesicles considering the average diameter (*d*) of 12.37 ± 0.29 μm after 6 h of thermal oligomerization (Figure 2b-i and c). After 12 h of thermal oligomerization, spherical micelles changed into branched structures with a branch width (*w*) of 1.39 ± 0.25 μm and a larger diameter (*d* = 62.19 ± 44.37 μm), as shown in Figure 2b-ii. Further thermal oligomerization resulted in a larger diameter (*d* = 131.75 ± 53.43 μm) with a smaller fiber width (*w* = 1.21 ± 0.22 μm) at 18 h oligomerization (Figure 2b-iii). After 24 h of thermal oligomerization, randomly coiled fibrous micelles were achieved with a greater diameter and a smaller fiber width (*d* = 223.93 ± 66.32 μm, *w* = 0.93 ± 0.10 μm) (Figure 2b-iv). Under a cross-polarized optical microscope, liquid crystalline oligomers demonstrate bright features in polarized optical microscope images, indicating nematic molecular alignments in both spherical and fibrous micelles (Figures S1 and S2). The oligomerization time-dependent growth of the liquid crystalline micelle is summarized in Figure 2c. The micelle’s diameter increases with the fibrous part’s decrement in width during the shape transition of liquid crystalline micelles from spherical to fibrous micelles under the thermal oligomerization process.

For further quantification, locally connected fractal dimension (*D*_F_) and lacunarity (λ) are analyzed, as shown in Figure 2d. A local complexity of patterns can be found in the locally connected fractal dimension. Its value displays the state of the dimension and how a pattern’s scale affects the level of detail. The fractal value of a 2D picture is between 1 and 2, which indicates that the fractal is in a state halfway between a line and an area. The increasing fractal dimension shows the development of a complex pattern. The higher fractal dimension indicates the formation of a detailed pattern [31,32]. Lacunarity is an image’s heterogeneity, which indicates its dispersity and homogeneity. For instance, the monodisperse sample has a low lacunarity value, and complicated pictures have a high lacunarity value [33]. The distribution of local connected fractal dimension was demonstrated in Figure 2b-v–viii, and results are plotted in Figure 2d. The fractal dimension decreased from 1.86 to 1.73 as the spherical micelle changed into the branched shape during 12 h oligomerization, which indicates increased linearity of the micelle. However, while micelles transition into fibrous shapes during further oligomerization, locally connected fractal dimension increased because entangled fibrous micelles merged into the area in 2D imagery. Lacunarity also increased during 12 h oligomerization due to the shape transition of the micelle and decreased as micelles changed in the fibrous branch.

**Figure 3:**
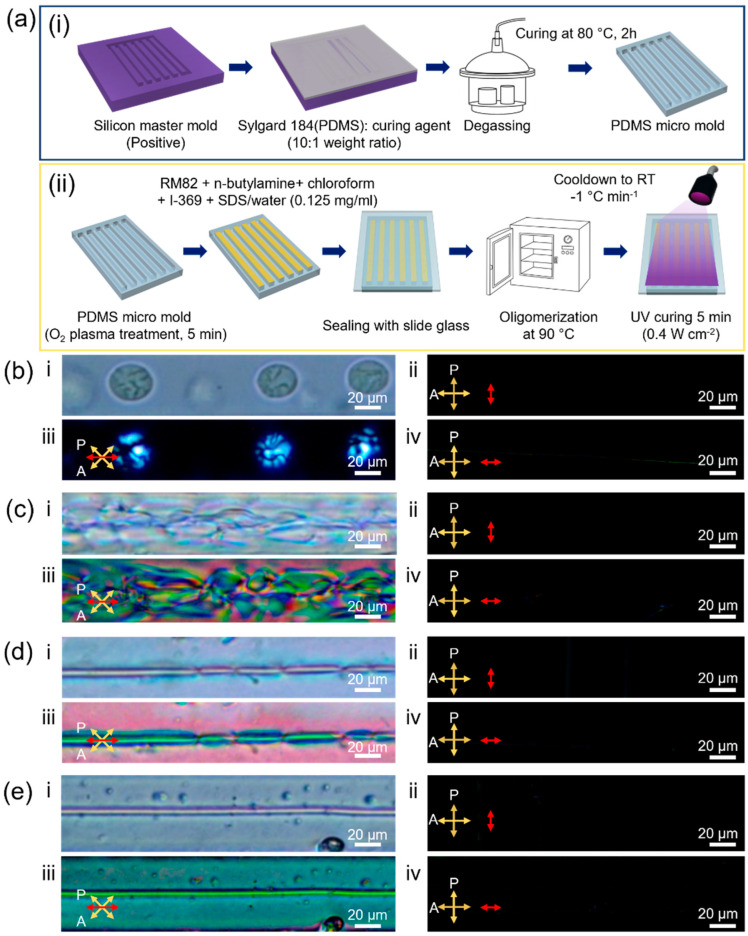
Spatial-confinement effects on shape changes in nematic liquid crystalline drops against thermal oligomerization times. (**a**) Schematic images of the construction of microchannel (**i**) and the thermal oligomerization procedure with spatial confinement (**ii**). (**b**–**e**) Shape transition of liquid crystalline oligomers from spherical to linear fiber geometry at different oligomerization times of (**b**) 6 h, (**c**) 12 h, (**d**) 24 h, and (**e**) 25 h. Scale bars are 20 μm. (**i**) Optical microscope image and (**ii**–**iv**) cross-polarized optical microscope images with a different directional offset of the micro-channel from the analyzer: (**ii**) 0°, (**iii**) 45°, and (**iv**) 90°. The red arrow indicates the long axis of the micro-channel.

To control the order of liquid crystalline monomers, directional ordering of liquid crystals in spatially confined condition must take place, as reported by Dong Ki Yoon and his coworkers [34]. Here, liquid crystalline micelles were thermally oligomerized in a micro-channel with a width of 50 μm, a depth of 20 μm, and a length of 7.5 mm (Figure S3) in the formation of spatial confinement during thermal oligomerization and shape transition. The microchannel was constructed with polydimethylsiloxane (PDMS) by soft lithography. The surface of the PDMS micro-channel was O_2_ plasma-treated for surface cleaning and wetting of the liquid crystalline emulsion on the PDMS mold. After pouring the liquid crystalline emulsion into the PDMS micro-channel, a glass slide was placed on the micro-channel as a superstrate to prevent the evaporation of water during thermal oligomerization. Following thermal oligomerization, liquid crystalline micelles were photopolymerized by irradiation of 365 nm ultraviolet light at an intensity of 0.4 W cm^−2^ for 5 min (Figure 3a). Even in the micro-channel, spherical micelles were formed until 6 h of thermal oligomerization (Figure 3b-i). The spherical geometry of the micelle indicates that the micelle does not experience spatial confinement at this point, as the size of the micelle is still much smaller than the micro-channel. Hence, the size of spherical micelles was uniformly controlled. Within the spherical micelle, however, fibrous rough textures can be observed. A cross-polarized microscope image revealed that liquid crystalline oligomers have nematic molecular alignments, as shown in Figure 3b-ii–iv. After 12 h of thermal oligomerization, liquid crystalline oligomers were anisotropically entangled along the channel and in the intermediate state between spherical and fibrous shapes (Figure 3c). This morphological transformation became more obvious following further thermal oligomerization. After 24 h of thermal oligomerization reaction, the liquid crystalline oligomer constructed almost linear fibers within the micro-channel with a few discontinuous defects (Figure 3d). Finally, high-aspect-ratio linear fiber was constructed without any discernable defects after 25 h of thermal oligomerization (Figure 3e). Along fiber direction, liquid crystalline oligomers were aligned, as evidenced from the bright cross-polarized microscope image when the fiber had a 45° offset angle with respect to both the polarizer and the analyzer (Figure 3c,d,e-iii) and dark image at 0° (ii)/90° (iv) in Figure 3c–e.

In a case where no such spatial limitations exist, the size of the micelle gradually increases from 12.37 ± 0.29 µm to 223.93 ± 66.32 µm as thermal oligomerization time increases, as shown in Figure 2c. Here, as the self-assembly of liquid crystalline monomers was performed in limited confinements of a micro-channel, the width of the micro-channel sets the limit for micelle size. A micro-channel width of 50 µm limits the size of a micelle, producing spatial confinement effects along the width direction. This anisotropic confinement induces the growth of the liquid crystalline oligomer perpendicular to the width direction of the micro-channel. Consequently, a single 5 µm-thick liquid crystalline oligomer fiber could be achieved, shown in Figure 3e, as opposed to the construction of multiple-layered micelles in a limited space.

**Figure 4:**
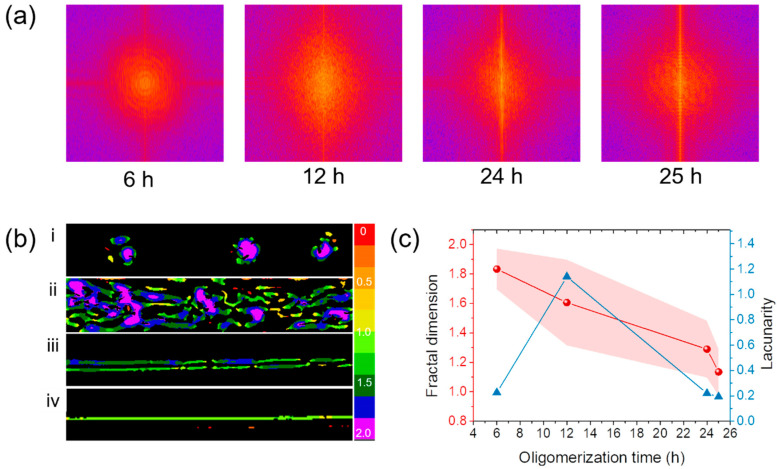
Spatial-confinement effects on shape change of nematic liquid crystal drop over oligomerization time. (**a**) Fast Fourier transform (FFT) images of spatially confined nematic liquid crystals at different oligomerization times. (**b**) Images of local connected fractal analysis at different oligomerization times: (**i**) 6 h, (**ii**) 12 h, (**iii**) 24 h, and (**iv**) 25 h. (**c**) Fractal dimension (●) and lacunarity (▲) of spatially confined grown nematic liquid crystals over oligomerization time.

Figure 4a shows the Fast Fourier Transform (FFT) images of spatially confined liquid crystalline micelles at different thermal oligomerization times. To visually examine the alignment direction and degree of the pattern, images were transformed into the frequency domain and analyzed by the FFT method. The areas presented in the converted image depend on frequency size. If a pattern was aligned along a single direction, the FFT image would show a series of points perpendicular to the direction of the original image. On the other hand, if the image has a random pattern, a circular region is produced in the FFT image. As thermal oligomerization time increased, the micelle became more fibrous and molecules aligned, as evidenced from the brighter vertical line pattern. After 25 h of thermal oligomerization, a straight fiber-shaped micelle was constructed (Figure 3e). Its FFT image shows the brightest and clear vertical center line. The local fractal dimension is visualized in Figure 4b. Then, fractal dimension and lacunarity were analyzed in Figure 4c. After 6 h of thermal oligomerization, the spherical liquid crystalline oligomers had a fractal dimension of 1.82. After 12 h of thermal oligomerization, the liquid crystalline structures are in an intermediary state between spherical and fibrous shapes. Despite changes in shapes, the fractal dimension slightly decreased to 1.61. Meanwhile, lacunarity significantly increased due to the heterogeneity of the liquid crystalline structures. When thermal oligomerization time increased to 24 h, both local fractal dimension and lacunarity significantly decreased for fibers with only a few defects. Finally, the fractal dimension further decreased to 1.13 with a single fiber shape after 25 h of thermal oligomerization. As the fractal dimension of 1 corresponds to a perfect line, 1.13 of the local fractal dimensions indicates the linearity of the fiber. In addition to the smallest local fractal dimension, the smallest value of lacunarity indicates the uniformity of the fiber polymerized in the spatially confined channel. 

In this study, we modified the architectures of liquid crystalline micelles from isotropic spherical to anisotropic fiber, which can reduce the surface tension by simply increasing thermal oligomerization time. When bottom-up molecular self-assembly was performed in a top-down micro-mold, we were able to manufacture single fibrous liquid crystal oligomers with a preferential molecular order along the micro-channel direction without the use of shears for wet-spinning or an electrical field for electrospinning. 

## 3. Materials and Methods

In 13.5 mg of chloroform, 4.5 mg of 1,4-bis-[4-(6-acryloyloxyhexyloxy)benzoyloxy]-2-methylbenzene (RM82, Synthon Chemicals, Wolfen, Germany) was dissolved with 0.5 mg of n-butylamine (Butylamine, Sigma Aldrich, St. Louis, MO, USA) and 2-benzyl-2-dimethylamino-1-(4-morpholinophenyl)-butanone-1 (I-369, Ciba, Basel, Switzerland). The liquid crystalline solution was mixed with an aqueous surfactant solution of 0.125 mg/mL of sodium dodecyl sulfate (SDS, Sigma Aldrich). Resultant suspensions were placed in a convection oven at 90 °C for thermal oligomerization for 6, 12, 18, and 24 h to observe the growth of liquid crystalline oligomers.

For the spatially confined growth experiment, a channel-shaped polydimethylsiloxane (PDMS) micro-mold was prepared with the following method. The PDMS precursor (Sylgard 184, Dow Corning, Midland, MI, USA) was mixed with a curing agent at a 10:1 (PDMS precursor: curing agent) weight ratio. The PDMS mixture was poured onto a positive silicon master mold featured with microchannels (50 μm width, 20 μm depth, and 7.5 mm length). The PDMS negative mold was prepared by placing it in a vacuum oven for thermal curing at 80 °C for 2 h. The PDMS microchannel was detached from the silicon mold after curing. The PDMS microchannel and slide glass were treated with air plasma for 5 min for surface cleaning. The liquid crystalline emulsion was poured into PDMS microchannels and sealed with a slide glass superstrate. The emulsion was thermally oligomerized in a 90 °C vacuum oven for 6, 12, 24, and 25 h. After thermal oligomerization, liquid crystalline oligomers were photo-polymerized upon exposure to 365 nm ultraviolet light at an intensity of 0.4 W cm^−2^ for 10 min. The photopolymerized liquid crystalline polymers were observed by a cross-polarized optical microscope (Nikon eclipse, Nikon, Tokyo, Japan) and analyzed by ImageJ and FracLac plugins.

## 4. Conclusions

In conclusion, we successfully polymerized liquid crystalline polymers in high-aspect-ratio fiber geometry by a hybrid approach. During the thermal oligomerization of liquid crystalline monomers, bottom-up molecular self-assembly is achieved within a micro-channel prepared by a top-down approach, followed by photopolymerization. Although most fiber production processes employ a high shear rate in a spinning process and high electrical voltage in an electrospinning process, this work suggests that spatial confinement can induce the self-assembly of directional fibrous micelle without applying large shear forces during the manufacturing process. Within high-aspect-ratio microfiber, we were able to generate nematic molecular alignments of the liquid crystalline polymers along the long axis of the fiber. This approach has potential in confinement-driven liquid crystalline colloidal systems for applications in non-conventional high-aspect-ratio fibers as well as soft actuators.

## Data Availability

Data are contained within the article.

## Supplementary Materials

The following supporting information can be downloaded at: https://www.mdpi.com/article/10.3390/molecules27175639/s1; Figure S1: Shape transition of liquid crystal oligomers from spherical to flower-shaped micelle (red arrow) over oligomerization time; Figure S2: Transmission optical microscope images and cross-polarized optical microscope image; Figure S3: Schematic images of micro-channel with dimensional information for length and depth of the micro-channel, optical microscope images and cross-polarized optical microscope image of the micro-channel.

## Sample Availability

Samples of the compounds are not available from the authors.
