# Supplementary material for: Confinement-Induced Fabrication of Liquid Crystalline Polymeric Fibers
Source: Molecules. 2022 Sep 1;27(17):5639. doi: 10.3390/molecules27175639 (PMC9458136; doi:10.3390/molecules27175639)
Supplement: Supplementary file 1 [file molecules-27-05639-s001.zip › molecules-1890561-SI.pdf]

molecules

Supplementary Materials

Confinement-Induced Fabrication of Liquid Crystalline Polymeric Fibers

Jae Gwang Kim [1], Jae Gyeong Lee [2] and Jeong Jae Wie [1,2,3,*]

[1] Department of Polymer Science and Engineering, Inha University, Incheon 22212, Korea
[2] Program in Environmental and Polymer Engineering, Inha University, Incheon 22212, Korea
[3] Department of Organic and Nano Engineering, Hanyang University, Seoul 04763, Korea
* Correspondence: wie@inha.ac.kr; jjwie@hanyang.ac.kr; Tel.: +82-32-860-7484

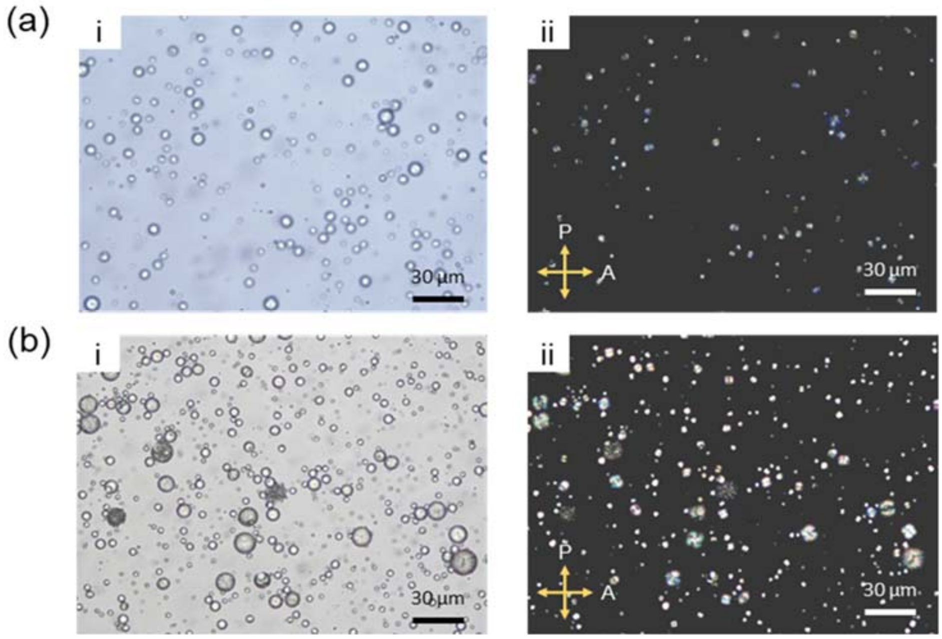

Figure S1. Shape transition of liquid crystal oligomers from spherical to flower-shaped micelle (red arrow) over oligomerization time. Optical microscope images (i) and cross polarized optical microscope image (ii) of LC emulsion after (**a**) 0 h and (**b**) 6 h of thermal oligomerization.

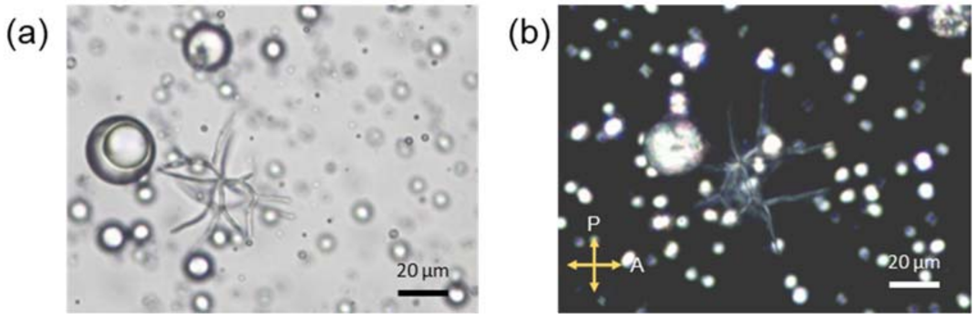

Figure S2. Transmission optical microscope images (**a**) and cross-polarized optical microscope image (**b**) for liquid crystalline oligomers after 12 h of thermal oligomerization.

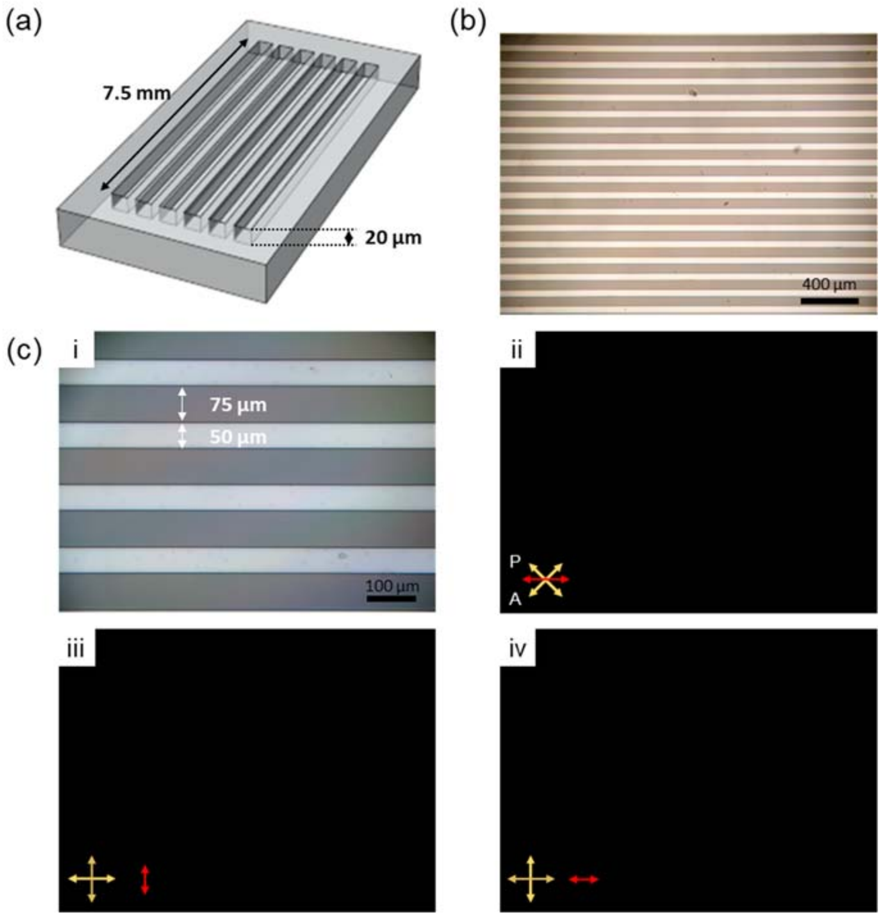

Figure S3. (**a**) Schematic images of micro-channel with dimensional information for length and depth of the micro-channel. (**b**) Optical microscope images and (**c**) cross-polarized optical microscope image of the micro-channel.
